# Efficacy of a live attenuated highly pathogenic PRRSV vaccine against a NADC30-like strain challenge: implications for ADE of PRRSV

**DOI:** 10.1186/s12917-021-02957-z

**Published:** 2021-07-31

**Authors:** Xin-xin Chen, Xinyu Zhou, Tengda Guo, Songlin Qiao, Zhenhua Guo, Rui Li, Qianyue Jin, Xiaofei Hu, Guangxu Xing, Ruiguang Deng, Bo Wan, Gaiping Zhang

**Affiliations:** 1 Key Laboratory of Animal Immunology of the Ministry of Agriculture, Henan Provincial Key Laboratory of Animal Immunology, Henan Academy of Agricultural Sciences, Zhengzhou, Henan People’s Republic of China; 2 College of Veterinary Medicine, Henan Agricultural University, Zhengzhou, Henan People’s Republic of China; 3 Jiangsu Co-innovation Center for Prevention and Control of Important Animal Infectious Diseases and Zoonoses, Yangzhou University, Yangzhou, Jiangsu People’s Republic of China

**Keywords:** Porcine reproductive and respiratory syndrome virus, Antibody-dependent enhancement, Vaccine protection

## Abstract

**Background:**

Porcine reproductive and respiratory syndrome virus (PRRSV) infection can cause severe reproductive failure in sows and respiratory distress in pigs of all ages, leading to major economic losses. To date, there are still no effective strategies to prevent and control PRRSV. Antibody-dependent enhancement (ADE), a phenomenon in which preexisting non-neutralizing antibodies or sub-neutralizing antibodies facilitate virus entry and replication, may be a significant obstacle in the development of effective vaccines for many viruses, including PRRSV. However, the contribution of ADE to PRRSV infection remains controversial, especially in vivo. Whether attenuated PRRSV vaccines prevent or worsen subsequent disease in pigs infected by novel PRRSV strains requires more research. In the present study, in vivo experiments were conducted to evaluate ADE under different immune statuses, which were produced by waiting different lengths of time after vaccination with a commercially available attenuated highly pathogenic PRRSV (HP-PRRSV) vaccine (JXA1-R) before challenging the pigs with a novel heterologous NADC30-like strain.

**Results:**

Piglets that were vaccinated before being challenged with PRRSV exhibited lower mortality rates, lower body temperatures, higher bodyweight gain, and lower viremia. These results demonstrate that vaccination with JXA1-R alleviated the clinical signs of PRRSV infection in all vaccinated groups.

**Conclusions:**

The obtained data indicate that the attenuated vaccine test here provided partial protection against the NADC30-like strain HNhx. No signs of enhanced PRRSV infection were observed under the applied experimental conditions. Our results provide some insight into the molecular mechanisms underlying vaccine-induced protection or enhancement in PRRSV.

## Background

Porcine reproductive and respiratory syndrome (PRRS), caused by PRRS virus (PRRSV), is a highly contagious disease in swine that is of great importance agriculturally. PRRSV is classified into two genotypes, PRRSV-1 (European type; prototype strain, Lelystad virus) and PRRSV-2 (North American type; the prototype strain, VR-2332). Because PRRSV leads to catastrophic economic losses worldwide swine industry worldwide every year, it has been the focus of much research since its initial emergence in the 1980s. In 2006, there was a particularly devastating outbreak in China of highly pathogenic PRRSV (HP-PRRSV) with a unique 30-amino-acid deletion in the PRRSV nonstructural protein 2 (nsp2) [1, 2]. In recent years, NADC30-like strains, which have a characteristic of unique discontinuous 131-amino-acid deletion in the Nsp2-coding region, have been the dominant epidemic strains in China [3]. Some NADC30-like PRRSV strains preferentially recombine with other PRRSV strains, such as HP-PRRSV strains and VR2332 [4, 5]. Consequently, PRRSV strains have extensive genetic and antigenic variation, and their frequent recombination leads to the emergence of diverse novel strains [3]. This contributes to the complexity of PRRSV and vaccine development and use. Several vaccines against PRRSV have been developed and are broadly used currently. Unfortunately, none of these commercially available vaccines can prevent PRRS. This might be partially the consequence of the field’s inadequate understanding of the role and mechanisms of antibody-dependent enhancement (ADE), which remain puzzling questions that affect the appropriate selection of immune strategies [6, 7].

ADE, first described in 1964, is a phenomenon in which preexisting non-neutralizing antibodies or sub-neutralizing concentrations of antibodies facilitate viral entry and replication [8]. The importance of ADE has been noted by prior studies. ADE can worsen disease severity and is a significant impediment to vaccine development and vaccination strategies. ADE has been reported to be of medical and veterinary importance in viruses from many different families. Among the viruses affected by ADE, the most notable are dengue virus (DENV), human immunodeficiency virus type 1 (HIV-1), Ebola virus, Zika virus (ZIKV), and PRRSV (owing to its veterinary importance) [9]. These viruses share some common characteristics, such as a tropism for myeloid cells, the establishment of persistent infection, and broad antigenic variability [10]. Myeloid cells bearing Fcγ receptors (FcγRs) mediate ADE through interacting with immunoglobulin (Ig) G antibody-virus complexes, thus increasing the attachment of virus to cells [11]. Some studies reported that complement receptor is also able to mediate the enhancement of West Nile virus (WNV) replication, as well as HIV and Ebola virus infection [12**–**14]. More intricately, several studies have observed that ADE also occurs between different viruses or different virus strains owing to cross-reactive antibodies [15]. For example, some antibodies directed against DENV or WNV are cross-reactive to ZIKV and can enhance ZIKV infection at specific concentrations in vitro; Furthermore, pretreatment with anti-ZIKV monoclonal antibody (mAb) or maternally acquired antibodies in vivo showed more severe symptoms and mortality in DENV-infected mice [16**–**18]. However, few studies have observed that preexisting anti-DENV antibodies enhance the pathogenesis of ZIKA. The mechanism underlying this clinical difference is still unclear. Therefore, extensive research is needed to clarify the mechanism behind the role of ADE in viral pathogenesis.

ADE of PRRSV infection was first described in 1993; the study reporting it found that viral replication was enhanced in fetuses inoculated with virus plus antibody as compared with that in fetuses inoculated with virus alone [19]. Yoon, et al. later reported that the viremia was elevated in pigs that were injected with sub-neutralizing amounts of PRRSV-specific IgG prior to virus challenge in vivo and also found that PRRSV-specific IgG enhanced the virus yields of heterologous strains [20, 21]. FcγRs, including FcγRI, FcγRIIb and FcγRIII, are involved in the ADE of PRRSV infection [22**–**25]. However, several reports have found no ADE in vivo with sub-neutralizing IgG, and modified live PRRSV vaccines provide partial cross-protection to heterologous field strains [26**–**29]. At present, there is insufficient in vivo evidences to definitely confirm if ADE plays an important role in PRRSV pathogenesis. The ADE of PRRSV infection might differ among different PRRSV strains and under different conditions. Previous in vitro studies showed that the enhancement of infection by anti-PRRSV sera was strongest at a dilution of 2^7^ [22]. Following the passive transfer of PRRSV-neutralizing antibodies, a higher serum concentration of PRRSV neutraling antibody titer at 1:32 induced full protection, but only in some young pigs, whereas a titer of 1:8 did not prevent PRRSV replication in the lungs or the dissemination of infection to other peripheral lymphoid tissues [29]. In light of these conflicting findings, additional research into the more details and underlying mechanisms of ADE in PRRSV infection is needed. The question of whether attenuated PRRSV vaccines worsen disease in pigs subsequently infected by novel strains needs to be addressed. In this study, in vivo experiments were designed to evaluate ADE effects in PRRSV infection through assessing the clinical manifestations, growth performance, viremia, and antibody response under different immune statuses, especially different antibody levels, implemented by challenging pigs with a novel heterologous NADC30-like PRRSV strain at different lengths of time after vaccination with a commercially attenuated HP-PRRSV vaccine.

## Results

### Clinical manifestations after JXA1-R inoculation and HNhx challenge

**Fig. 1:**
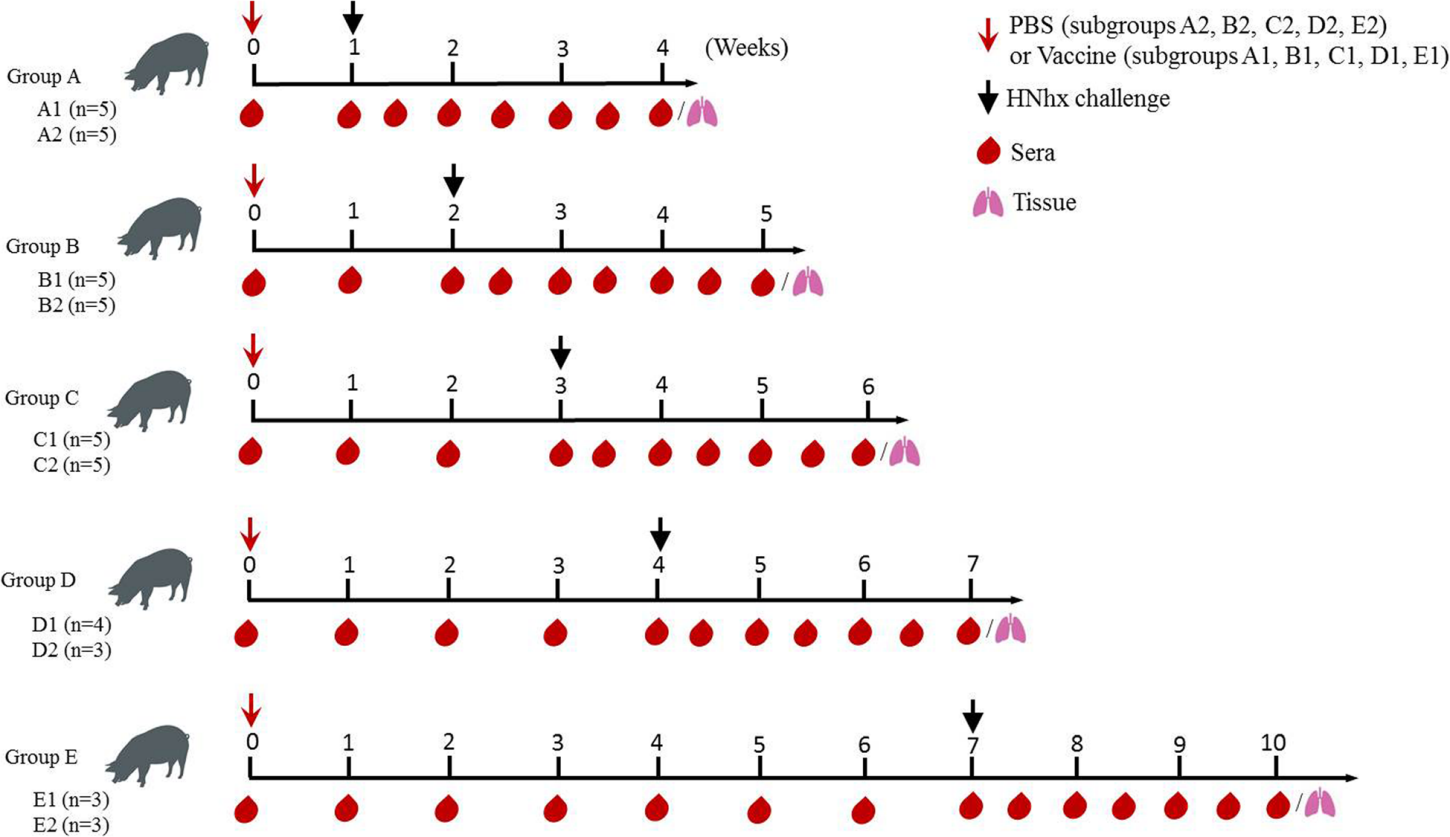
Vaccination and challenge strategies. At week 0, 4-week-old piglets were inoculated with attenuated PRRSV vaccine at the indicated dose via an intramuscular injection in accordance with the manufacturer’s instructions or with PBS as a mock-vaccination control (red stealth arrow). Then, A PRRSV strain HNhx challenge was then performed in the piglets of each group at the indicated week post-vaccination (black arrow). Sera and lung tissues were collected at her marked timepoints

To simulate the clinical situation and acquire different immune statuses in pigs before PRRSV challenge, the vaccination and challenge strategy was designed as illustrated in Fig**.** 1. HNhx challenge was implemented at different lengths of time after vaccination with JXA1-R. All pigs were carefully monitored to observe clinical signs of PRRS, such as cough, depression, sneezing and anorexia. As expected, none of the piglets in the negative-control (mock-challenge) group exhibited clinical symptoms of PRRS. After JXA1-R inoculation, a slight decrease of appetite was observed in all vaccinated piglets (subgroups A1, B1, C1, D1, and E1). After HNhx challenge, most piglets showed obvious PRRSV-specific clinical signs, such as cyanosis or erythema of the skin over the ears, shivering, inappetence, and fever.

### JXA1-R-inoculated piglets had a higher survival rate

**Fig. 2:**
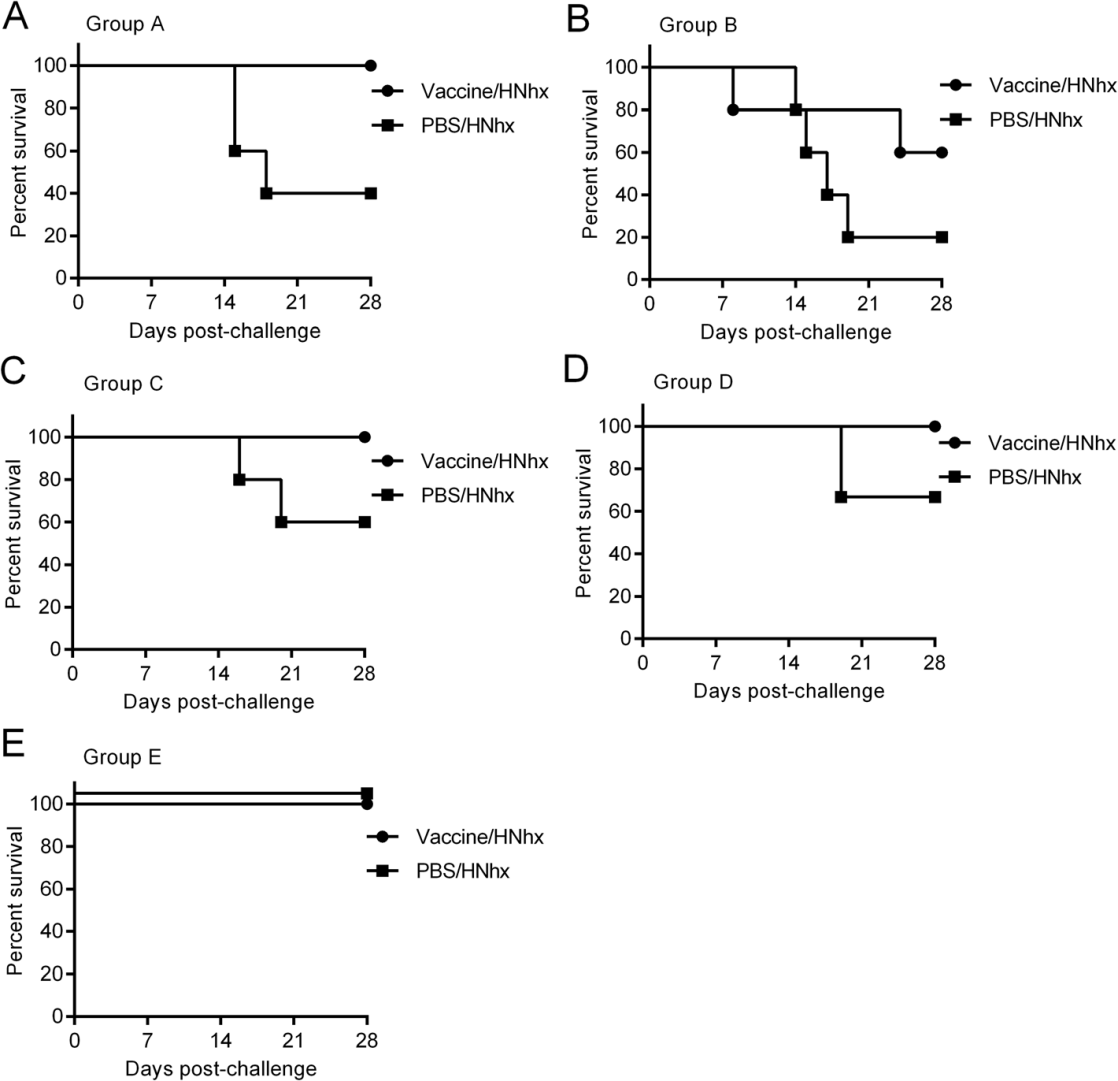
Survival rates of piglets in each group

In group A, composed of piglets vaccinated with JXA1-R (subgroup A1) or phosphate-buffered saline PBS (subgroup A2) and then challenged with HNhx at 7 days post-vaccination (dpv), three out of five mock-vaccinated piglets in subgroup A2 died before 28 days post-challenge (dpc), whereas all the vaccinated piglets in subgroup A1 survived (Fig**.** 2). In group B, composed of piglets vaccinated with JXA1-R (subgroup B1) or PBS (subgroup B2) and then challenged with HNhx at 14 dpv, four of five mock-vaccinated piglets in subgroup B2 died before 28 dpc, whereas two of five vaccinated piglets in subgroup B1 died. In group C, composed of piglets vaccinated with JXA1-R (subgroup C1) or PBS (subgroup C2) and then challenged with HNhx at 21 dpv, two of five mock-vaccinated subpiglets in group C2 died before 28 dpc, whereas all five vaccinated piglets in subgroup C1 survived. In group D, composed of piglets vaccinated with JXA1-R (subgroup D1) or PBS (subgroup D2) and then challenged with HNhx at 28 dpv, one of three mock-vaccinated piglets in subgroup D2 died before 28 dpc, whereas all four vaccinated piglets in subgroup D1 survived. In group E, composed of piglets vaccinated with JXA1-R (subgroup E1) or PBS (subgroup E2) and then challenged with HNhx at 49 dpv, all piglets survived until the end of the study.

### Mock-vaccinated piglets exhibited much higher and longer clinical fevers after HNhx challenge

**Fig. 3:**
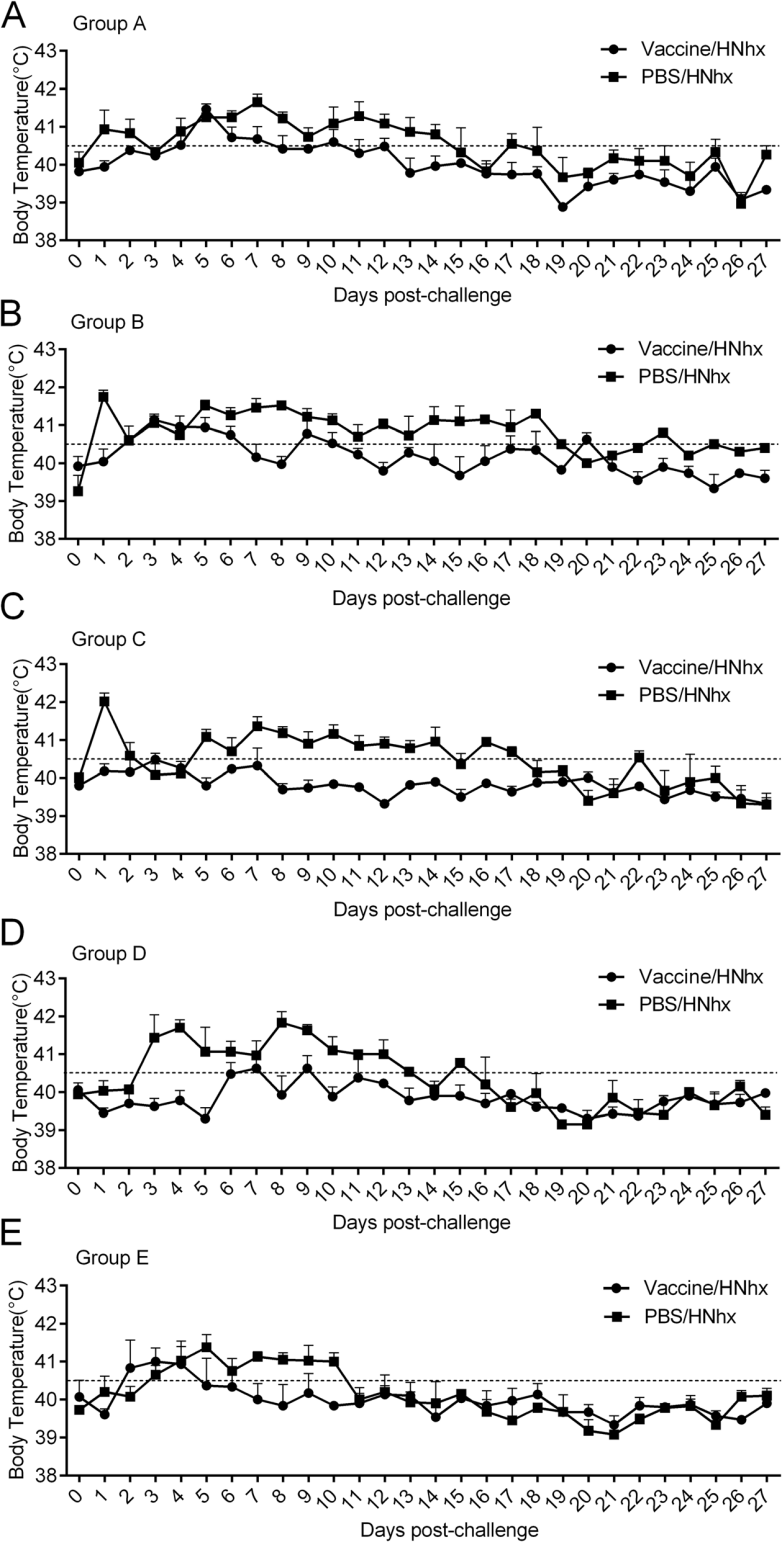
Daily rectal temperature of piglets after PRRSV challenge. A rectal temperature of over 40.5 °C, indicated with dotted lines, was defined as high fever. Data are shown as the mean ± S.E.M

The mock-vaccinated, PRRSV-challenged piglets (PBS/HNhx) in subgroups A2, B2, and C2 developed a sharp high fever (above 40.5 °C) at 1 dpc, and this fever lasted for approximately 14, 18, and 17 days, respectively, except for few days below 40.5 °C. In contrast, the piglets in subgroup D2 exhibited a high fever from 3 to 13 dpc, and piglets in subgroup E2 exhibited a high fever from 2 to 10 dpc. Thus, the duration of fever in mock-vaccinated piglets was significantly prolonged with age after PRRSV challenge (Fig**.** 3). More importantly, almost all mock-vaccinated pigs in groups A–E exhibited a higher rectal temperature compared with vaccinated piglets at each single day with a high fever. Thus, vaccination with JXA1-R prevented piglets from having a fever caused by PRRSV infection.

### JXA1-R-inoculated piglets exhibited relatively good growth performance

**Fig. 4:**
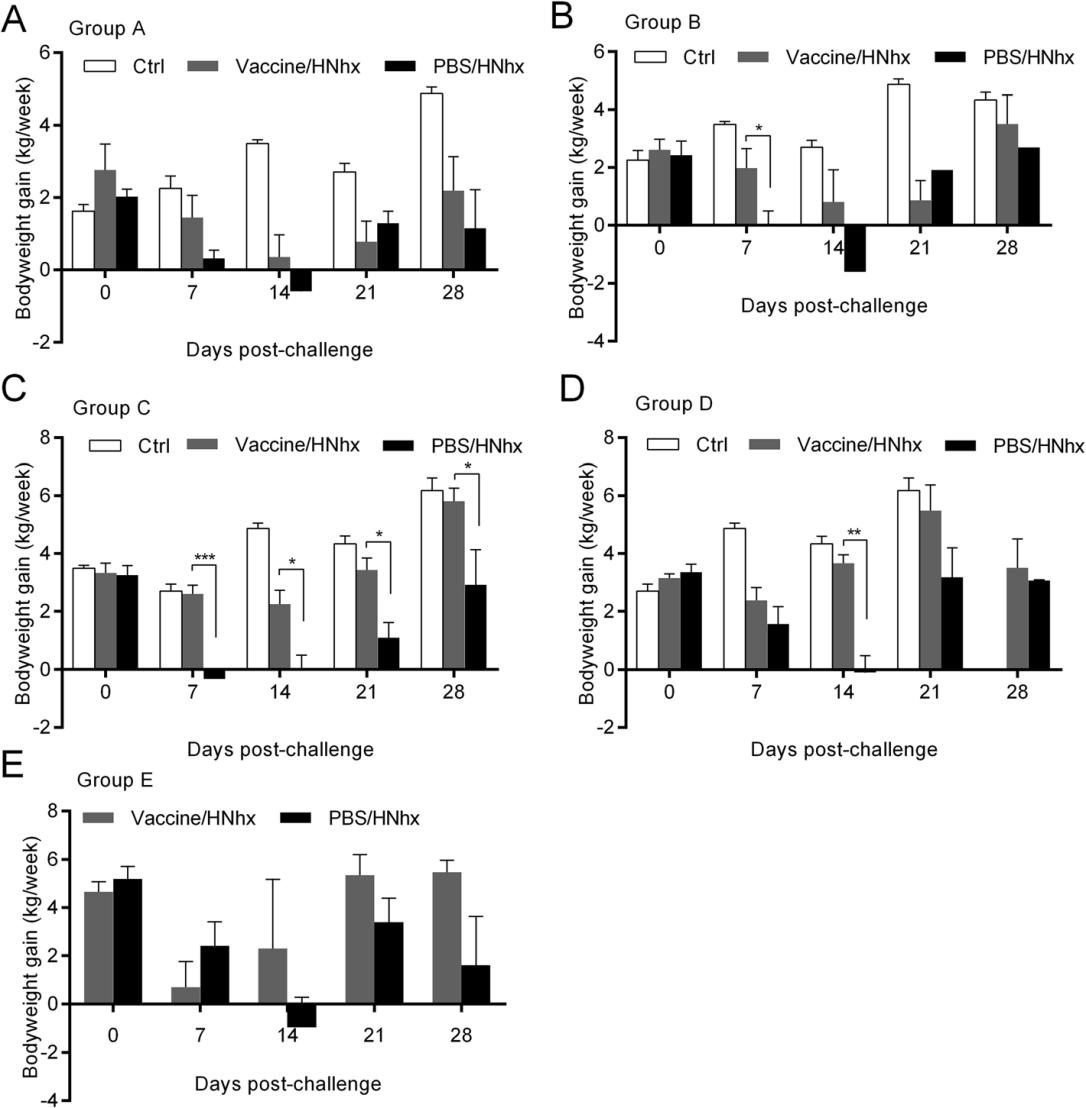
Bodyweight gain of piglets after PRRSV challenge. The bodyweight gain of each piglet after PRRSV challenge was calculated. Data are shown as the mean ± S.E.M. Differences between groups were assessed using a Student’s *t*-test; statistical significance is denoted as follows: *p < 0.05, **p < 0.01, and ***p < 0.001

The bodyweight of each piglet was assessed weekly. At week 0, the bodyweight gain of piglets in the negative control, Vaccinated/HNhx, and PBS/HNhx groups were not significantly different. After HNhx challenge, regardless of vaccination history, the bodyweight gain of HNhx-challenged piglets was lower than that of control (mock-challenged). However, JXA1-R-inoculated piglets had relatively higher bodyweight gain compared with mock-vaccinated piglets, especially at 7 and 14 dpc (Fig. 4).

### Mock-vaccinated piglets exhibited higher virus titers in their sera and tissues

**Fig. 5:**
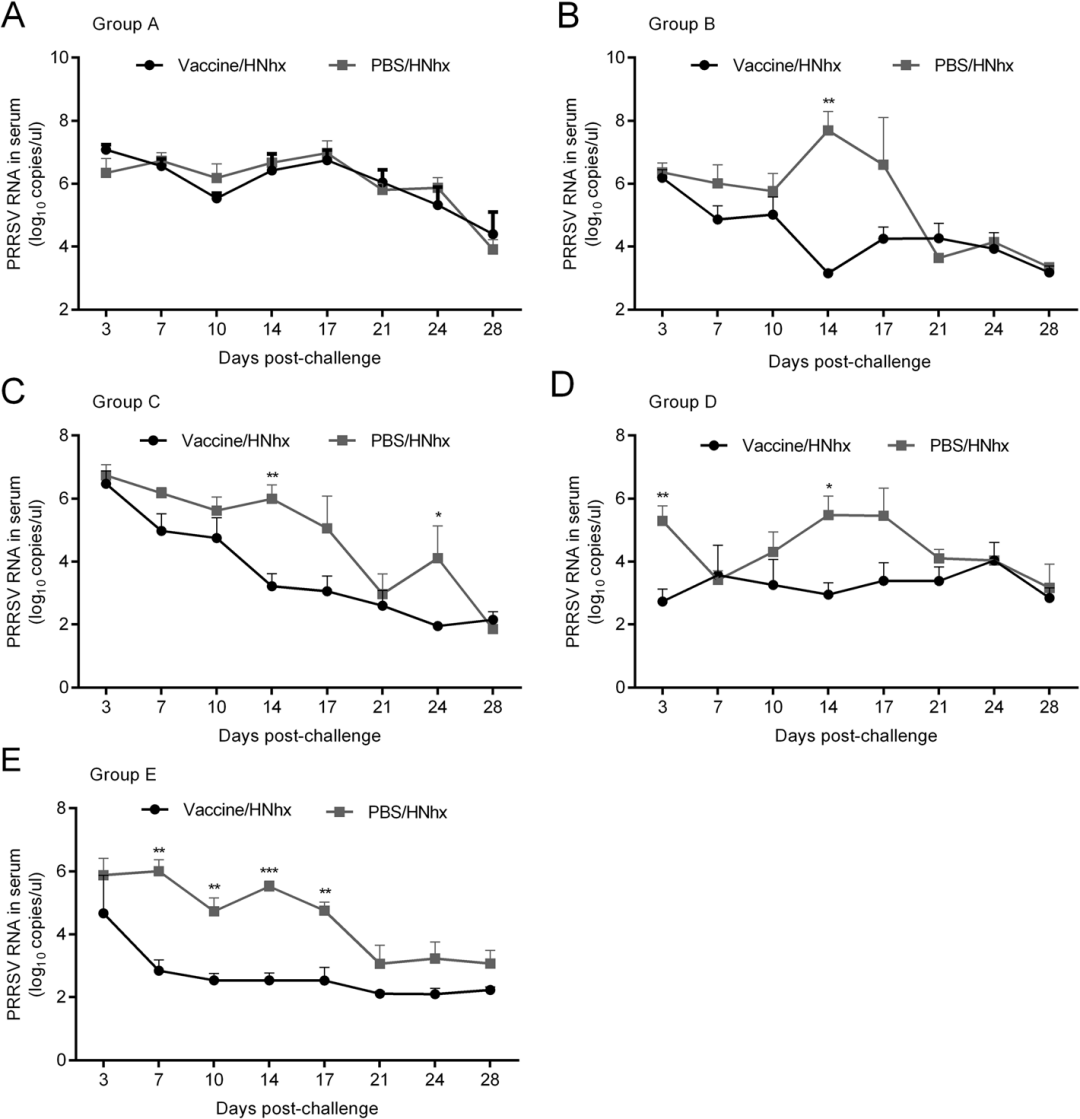
Viremia of piglets after PRRSV challenge. RNA in sera collected from each piglet in the different groups was extracted and subjected to absolute quantitative real-time PCR for PRRSV detection. Differences between groups were assessed using a Student’s *t*-test; statistical significance is denoted as follows: *p < 0.05, **p < 0.01, and ***p < 0.001

Blood samples were collected from each piglets at 0, 3, 7, 10, 14, 17, 21, 24 and 28 dpc for viremia detection using absolute quantitative real-time PCR with primers targeting the nsp2 region. There was no difference in the PRRSV RNA copy numbers between vaccinated and mock-vaccinated piglets in group A. But the mock-vaccinated piglets in groups B, C, and D exhibited much higher levels of viremia, which was mainly reflected at timepoints prior to 21 dpc (Fig. 5).

**Fig. 6:**
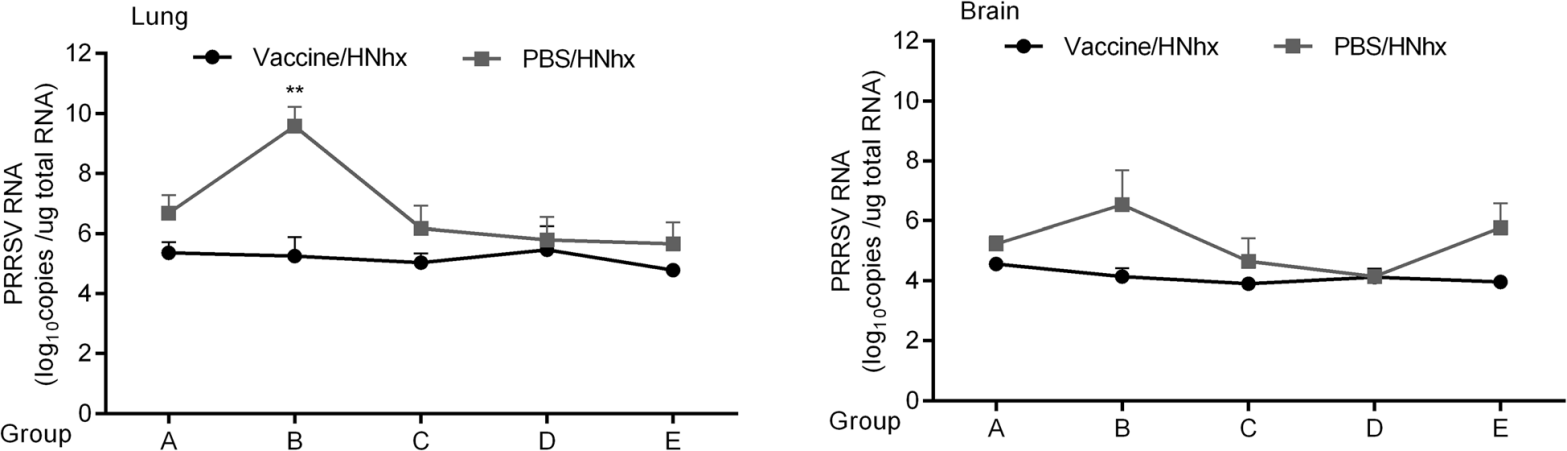
Viral loads in piglet tissue samples. Tissue samples were collected from piglets that were euthanized when they were dying or at the end of studies. **A**, **B** The viral RNA in the lungs (A) and brains (B) from each pig in the different groups was then detected using absolute quantitative real-time PCR. Differences between groups were assessed using a Student’s *t*-test; statistical significance was denoted as follows: *p < 0.05, **p < 0.01, and ***p < 0.001

The virus RNA copy numbers were also assessed in tissue samples of the lungs and brain. The virus titer was significantly higher in samples from the mock-vaccinated subgroup B2 than in those from the vaccinated subgroup B1. Whereas the virus titer showed no obvious difference between subgroups D1 and D2 (Fig. 6).

### JXA1-R-inoculated piglets developed higher levels of PRRSV-specific antibodies

**Fig. 7:**
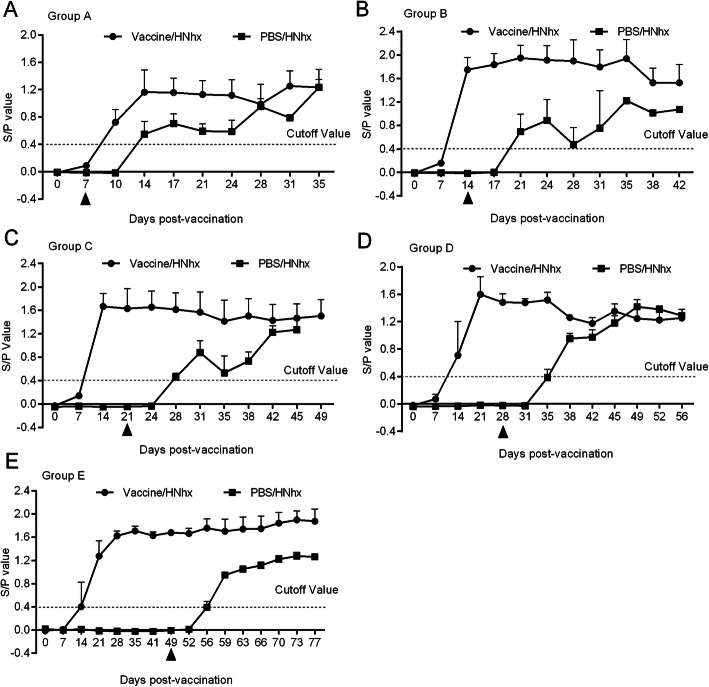
Serological response. PRRSV-specific antibodies in the collected sera were detected by IDEXX ELISA kits. The threshold for seroconversion was set at a sample-to-positive (s/p) ratio of 0.4. Triangle (▲) indicate the timepoints of HNhx challenge

The humoral immune responses in piglets were examined through measuring PRRSV-specific antibodies using an IDEXX ELISA kit. PRRSV-specific antibodies were positively detected in vaccinated piglets from 14 dpv, indicating that the commercial JXA1-R vaccine used here was able to induce an antibody response. **T**he PRRSV-specific antibody levels in these piglets remained relatively stable after 14 dpv (Fig. 7A–C) or 21 dpv (Fig. 7D&E), regardless of whether the piglets were infected with HNhx. Thus, viral infection alone (PBS/HNhx subgroups) can also induce PRRSV-specific antibody production.

## Discussion

PRRS remains a major challenge in the pig industry. PRRSV is constantly evolving and cause new outbreaks and epidemics with much stronger virulence [30]. HNhx, isolated by our lab, is the result of a recombination between the NADC30 strain and the HP-PRRSV vaccine strain JXA1-P80 in Nsp4 (nt 5261) to Nsp9 (nt 7911) [31]. Whether currently available attenuated PRRSV vaccines prevent or worsen disease from subsequent PRRSV infection requires additional research. A few studies have investigated the effects of attenuated PRRSV vaccines against NADC30-like strains. Some attenuated vaccines, like modified-live virus (MLV) vaccines, derived from classical PRRSV (VR2332) or HP-PRRSV, were reported to confer cross protection to the prevailing NADC30-like strain [32**–**34]. In the present study, to use the difference in antibody levels and immune status of piglets at different timepoints after vaccination, piglets were challenged with novel HNhx at different lengths of time (1, 2, 3, 4, and 7 weeks) after vaccination. The results show that vaccinated piglets had lower mortality rates (Groups A–D, Fig. 2), lower body temperature, and higher bodyweight gain compared with mock-vaccinated piglets, indicating that vaccination with JXA1-R alleviated the clinical signs in all groups. Although the tested vaccine provided partial protection against the NADC30-like strain HNhx, its cross-protection was limited, which is consistent with previous studies [35]. It has no significance in pig production, given that vaccinated groups grew much slower than pigs in negative control group and gained much lower weight than that of control piglets. Besides, among the piglets in the vaccinated subgroups, two in group B died at 8 dpc and 24 dpc, respectively (Fig. 2), and vaccinated piglets in subgroup B1 showed the longest duration of high fever starting from 2 dpc to 6 dpc (Fig. 3).

Vaccines are usually effective strategies for virus control. Since the emergence of PRRSV, several different kinds of vaccines have been developed and widely adopted in the field, the most common of which are inactivated vaccines and live attenuated vaccines. However, inactivated vaccines against PRRSV cannot elicit a strong immune response, and live attenuated vaccines usually provide effective homologous protection but limited protection against heterologous strains, owing to the vast genetic diversity and high mutation rate of PRRSV [7, 19]. Additionally, ADE is one of the most important factors hampering the development of efficacious vaccines for many viruses. For viral infections affected by ADE, vaccination runs the risk of contributing to an increased sensitivity to virus infection, as has been reported in many viruses across different viral families, such as DENV, HIV, and coronavirus [36, 37]. Recent studies found no evidence for a role of antibodies in vaccination-induced enhancement of PRRSV [38].

Although PRRSV infection can elicit an antibody response at 7–9 days post infection (dpi), the generated antibodies lack neutralizing ability against PRRSV in vitro [39], and the passive transfer of these early serum antibodies may enhance infection [29]. Neutralizing antibodies typically appear after 28 dpi [39]. Thus, given the lack of ADE observed in A–D, we expanded our experiment by adding group E, in which piglets were challenged with PRRSV at 49 dpv. Similar to those in groups A–D, vaccinated piglets in group E had lower body temperatures and higher bodyweight gain compared with mock-vaccinated piglets. Even when the PRRSV challenge occurred well after neutralizing antibodies are usually generated, no ADE was observed under our experimental conditions. However, unlike those in groups A–D, whether vaccinated or not, all piglets in group E survived, regardless of their PRRSV vaccination status (Fig. 2). This higher survival rate might be because these pigs in Group E were older at the time of PRRSV challenge compared with those in the other groups. The finding that innate innate immune resistance increased with ageis consistent with previous studies [40].

## Conclusions

Together, our results demonstrate that piglets vaccinated against PRRSV had lower mortality rates, lower body temperature, higher bodyweight gain, lower viremia, and higher levels of PRRSV-specific antibodies than piglets that did not receive the vaccination, indicating that vaccination with JXA1-R slightly alleviated the clinical signs in all groups. No ADE was observed in vivo under our experimental conditions. The data from this study provide some insight into the molecular mechanisms underlying vaccine induced protection or enhancement in PRRSV.

## Methods

### Cells, virus and vaccines

Porcine alveolar macrophages (PAMs) were obtained from lung lavage of 4- to 6-week-old healthy piglets that were free of PRRSV, and these cells were maintained in Roswell Park Memorial Institute 1640 medium (RPMI 1640) with 10% heat-inactivated fetal bovine serum (FBS), 100 U/ml penicillin, and 100 μg/ml streptomycin at 37 °C in a humidified atmosphere with 5% CO_2_.

HNhx (GenBank accession number KX766379), a NADC30-like strain, was isolated by our laboratory in 2017 [31]. HNhx was propagated and titrated on PAMs. The viral titer was determined by applying the Reed-Muench method and designated by the tissue culture infective dose 50% (TCID_50_/ml). Viruses were stored at − 80 °C until use.

JXA1-R is a commercially available HP-PRRSV attenuated vaccine that was purchased from Pulike Biological Engineering Co., Ltd.

### Animals and experimental design

Forty-six 3-week old weaned piglets that were free of PRRSV, pseudorabies virus, porcine circovirus, and classical swine fever virus were obtained from Henan Huayang Agriculture and Animal Husbandry Co., Ltd. These piglets were acclimated to our facilities for 1 week prior to their use in our study. They were randomly divided into six groups, designated as control and groups A–E (the treatment of piglets in each group is shown schematically in Fig. 1). Each group (A–E) was further divided in two subgroups: A1/A2, B1/B2, C1/C2, D1/D2, and E1/E2, respectively. Each subgroup was raised separately in animal facilities. The control group (*n* = 3) received PBS as a negative control. At week 0 (4 weeks old of age), pigs in the Vaccine/HNhx subgroups (A1, *n* = 5; B1, *n* = 5; C1, *n* = 5; D1, *n* = 4; and E1, *n* = 3) were intramuscularly immunized with a single dose of JXA1-R, and pigs in the PBS/HNhx subgroups (A2, *n* = 5; B2, *n* = 5; C2, *n* = 5; D2, *n* = 3; and E2, *n* = 3) were similarly inoculated with PBS. All piglets in groups A–E were then subjected to challenge with HNhx (2 × 10^5^ TCID_50_/piglet) administered by intranasal inoculation at 7 (group A), 14 (group B), 21 (group C), 28 (group D), or 49 (group E) dpv.

### Clinical observation

The health of each piglet was carefully monitored. After vaccination and challenge, pigs were examined daily until the end of the study for their rectal temperature and clinical signs, including depression, cough, diarrhea, dyspnea, and shivering. Their survival rates were calculated. Growth performance was assessed by bodyweight gain per week, which was calculated by recording the bodyweight of each piglet. Blood samples were collected weekly between vaccination and challenge, then twice weekly after challenge. When piglets were dying or reached the end of the study, they were euthanized via an intravenous injection with an overdose of sodium pentobarbital (100 mg/kg bodyweight). Piglets were then necropsied, and tissue samples were collected.

### Serology

Serum was obtained from collected blood samples and tested for PRRSV-specific antibody by using a commercially available PRRSV antibody test kit (IDEXX PRRS X3 Ab test, IDEXX Laboratories Inc., Westbrook, Maine, USA) following the test procedure. The cut-off value of the sample-to-positive (S/P) ratio was set at 0.4 in accordance with the manufacturer’s instructions. Serum samples with an S/P ratio of ≥0.4 were considered to be positive for PRRSV-specific antibodies.

### Detection of virus in sera and tissue

To quantify the amount of PRRSV in sera and tissue, absolute quantitative real-time PCR was used. Primers targeting the nsp2 region were designed for standard plasmids construction and used to differentiate between HNhx and JXA1-R (Table 1). Specifically, TRIzol LS (Invitrogen) was used to extract total RNA from serum samples and TRIzol (Invitrogen) was used to extract total RNA from tissues after their homogenization, following the manufacturer’s instructions. The extracted RNA was subjected to reverse transcription PCR using PrimeScript RT Master Mix (TaKaRa) in accordance with the manufacturer’s instructions. Real-time PCR was performed with a FastStart Universal SYBR Green Master (Rox) Kit (Roche) on a 7500 fast real-time PCR system (Applied Biosystems).

**Table 1:** Primers and probe used in cloning and Quantitative RT-PCR

Name	Sequence (5′-3′)
JXA1-R (clone)	F: TGTCCTGGAAGAATATGGG R: GCAATCGGATCTGACCTT
JXA1-R (RT-PCR)	F: AACTAACCAACACCCAGGCG R: CGGGTAGCTTTTGACCCAAG
JXA1-R Probe	FAM-CGACTTCAGAAATGATGGCCTGGGCGG-BHQ-1
HNhx (clone)	F: GGTGGTTCCTTCCATTCTCC R: CTCTGCGGCAACGTCAAA
HNhx (RT-PCR)	F: GCTGAAGCCGTCACCGATA R: TTCATTCCTCCCACCTGCTG
HNhx-Probe	CY5-CGTCAACCCCTGTGCCCGCACCAC--BHQ-2

*F* Forward primers, *R* Reverse primer

### Statistical analysis

Statistical analysis was performed by conducting *t*-tests using GraphPad Prism software 7 (San Diego, CA). A *p*-value of < 0.05 was considered to indicate a statistically significant difference.

## Data Availability

The datasets supporting the conclusions of this article are included within the article.

## Abbreviations

PRRSV: Porcine reproductive and respiratory syndrome virus
ADE: Antibody-dependent enhancement;
HP-PRRSV: Highly pathogenic PRRSV
nsp2: Nonstructural protein 2
DENV: Dengue virus
HIV-1: Human immunodeficiency virus type 1
ZIKV: Zika virus
FcγRs: Fcγ receptors
PAMs: Porcine alveolar macrophages
RPMI 1640: Roswell Park Memorial Institute 1640 medium
FBS: Fetal bovine serum
TCID_50_: Tissue culture infective dose 50%
